# Bone Marrow Transplantation Concurrently Reconstitutes Donor Liver and Immune System across Host Species Barrier in Mice

**DOI:** 10.1371/journal.pone.0106791

**Published:** 2014-09-05

**Authors:** Ziping Qi, Lu Li, Xuefu Wang, Xiang Gao, Xin Wang, Haiming Wei, Jian Zhang, Rui Sun, Zhigang Tian

**Affiliations:** 1 Department of Immunology, School of Life Sciences, University of Science and Technology of China, Hefei, Anhui, China; 2 Hefei National Laboratory for Physical Sciences at Microscale, Hefei, Anhui, China; 3 Model Animal Research Center, Nanjing University, Nanjing, China; 4 Institute of Biochemistry and Cell Biology, Shanghai Institute for Biological Sciences, Chinese Academy of Sciences, Shanghai, China; 5 School of Pharmaceutical Sciences, Shandong University, Jinan, China; 6 Collaborative Innovation Center for Diagnosis and Treatment of Infectious Diseases, Hangzhou, Zhejiang, China; Rutgers - New Jersey Medical School, United States of America

## Abstract

Liver immunopathologic mechanisms during hepatotropic infection, malignant transformation, and autoimmunity are still unclear. Establishing a chimeric mouse with a reconstituted liver and immune system derived from a single donor across species is critical to study regional donor immune responses in recipient liver. Using a strain of mice deficient in tyrosine catabolic enzyme fumarylacetoacetate hydrolase (*fah* ^-*/*-^) and bone marrow transplantation (BMT), we reconstituted the donor's hepatocytes and immune cells across host species barrier. Syngeneic, allogeneic or even xenogeneic rat BMT rescued most recipient *fah^-/-^* mice against liver failure by donor BM-derived FAH^+^ hepatocytes. Importantly, immune system developed normally in chimeras, and the immune cells together with organ architecture were intact and functional. Thus, donor BM can across host species barrier and concurrently reconstitutes MHC-identical response between immune cells and hepatocytes, giving rise to a new simple and convenient small animal model to study donor's liver immune response in mice.

## Introduction

The liver diseases caused by hepatitis B virus and hepatitis C virus infection are among the most important human health problems [1]. The immunopathogenesis of virus infection and the development of antiviral drugs are hampered by the lack of suitable mouse models for both pathogens, because the mouse cannot be infected by HBV or HCV. Although chimpanzees are susceptible to both infections, their usage is limited by cost, availability, and ethical considerations [2], [3]. Other surrogate hepatotrophic viruses that infect ducks [4], woodchucks [5], and ground squirrels [6] have been widely used to study virus biology, however, they suffer from two important limitations: (1) on the microbial side, surrogate viruses are genetically divergent from highly restricted human counterparts; and (2) on the host side, the immunological studies in genetically outbred and immunologically uncharacterized hosts are difficult. HBV-transgenic mice have provided invaluable information on immunopathogenesis of HBV, whereas transgenic mice are immunologically tolerant to transgene products [7]. Hydrodynamic transfection of the mouse liver by the HBV genome has also been reported to study HBV immunobiology [8], but it does not support bona fide viral infection. Therefore, a robust and reproducible mouse model of HBV or HCV infection is desperately needed, and studies based on chimeric mice appear to be the most promising.

Establishing a chimeric mouse with a reconstituted liver and immune system derived from a single donor is critical to study MHC-restricted immune responses against pathogen-infected, transformed or autoimmune hepatocytes in a physiologic setting. In the separate experiments [9]–[12], it was reported that donor hematopoietic stem cell (HSC) can differentiate into not only immune cells but also hepatocytes, which greatly helps understanding the maturation of donor immune system, and virus-infected donor hepatocytes, respectively; however, from our knowledge, no experiment was practically economically carried out to exhibit the donor immune response against their own hepatocyte-presented antigens because lacking chimeras mouse with a MHC-matched response between immune cells and hepatocytes which needs a dual reconstitution from a single donor.

A widely accepted method to construct chimeras with a complete donor immune system is hematopoietic stem cell (HSC) transplantation into lethally irradiated or immunodeficient recipients, such as NOD-*scid-Il2rγ* ^—/-^ (NOG) [13], [14] or BALB/c-*Rag2* ^-/—^ *Il2rγ* ^—/-^ mice [10]. These two kinds of mice were highly deficient in immune system, leaving the space for donor's HSC engraftment, especially for the development of human immune system (HIS) in humanized mouse. Chimeras with donor-derived hepatocytes can also be created by transplanting exogenous hepatocytes or embryonic stem cells, like in the uroplasminogen-activator (uPA) transgenic [15] or fumarylacetoacetate hydrolase (FAH)-deficient models [16]. Both uPA transgenic mouse and FAH deficient mouse suffer from progressive liver failure, so that donor's hepatocytes could engraft and repopulate in recipient mouse more easily. However, neither the immune- nor liver-reconstituted chimera alone is sufficient to further evaluate the interaction between the immune system and the pathogen-infected or inflamed liver organs.

There is a significant need for a model system, with MHC-identity between donor immune cells and pathogen-targeting organs, to further investigate the pathology, immune correlates, and mechanisms of highly specialized pathogens like HBV, HCV and malaria (at liver stage). To avoid the potential complication from histocompatibility, hematopoietic and hepatic (or any other organ origin) progenitors had better to be from the same donor. It was recently reported that HSC may also differentiate into hepatocytes in bone marrow transplanted (BMT) mice [9], [17]–[20]. We therefore hypothesized that donor HSCs may concurrently differentiate into immune cells and hepatocytes in recipients that have open tissue space, which will greatly benefit exploiting the donor's MHC-restricted interaction between immune cells and hepatocytes.

Here, using *fah-*deficient mice and BMT, we reconstituted donor hepatocytes and immune cells across host species barrier. All recipient *fah^-/-^* mice survived without NTBC feeding at least 5 months after syn-, allo- and xeno- BMT (rat), and donor BM-derived hepatocytes were detected in liver sections. Importantly, donor immune systems developed normally in MHC-identical chimeras, and the immune cells together with organ architecture were intact and functional. Thus, donor BM can across host species barrier and concurrently reconstitutes MHC-identical response between immune cells and hepatocytes. Thus, this method gives rise to a new simple and convenient small animal model to study donor's liver immune response across host species barrier in mice.

## Materials and Methods

### Mice

C3H/HeJSlac mice and Sprague-Dawley (S.D.) rats were purchased from the Shanghai Experimental Center, Chinese Science Academy (Shanghai, China). EGFP-transgenic mice were purchased from the Model Animal Research Center (Nanjing, China), who originally obtained them from the Jackson Laboratory (Bar Harbor, ME, USA). *Fah^-/-^* mice (a gift from Xin Wang) were normally treated with NTBC in the drinking water at a concentration of 7.5 mg/L.

### Ethics Statement

All animals were housed in a specific pathogen-free facility and used according to the animal care regulations of the University of Science and Technology of China. The study was approved by the Local Ethics Committee for Animal Care and Use at University of Science and Technology of China (Permit Number: USTCACUC 1201009). Surgery mice were anesthetized by intraperitoneal injection of 30 µg/g body weight sodium pentobarbital, and the surgeries were performed by skillful experimenter. Sacrificed animals were euthanized by CO_2_, and all efforts were made to minimize suffering. For survival study, mice were monitored daily. A humane endpoint was always used, and moribond animals were euthanised by CO_2_. The clinical criteria used to determine the endpoint include: a). Rapid or progressive weight loss. b). Rough hair coat/unkempt appearance. c). Hunched posture, dehydration, and hypothermia. d). Lethargy or persistent recumbency, immobility. e). Decreased food or water intake. f). Opacity eyes, lack of responsiveness to manual stimulation.

### BMC harvest and transplantation

BMCs were harvested by flushing long bones of mice or rats with 1640 medium (Gibco, New York, USA) and then washed with phosphate-buffered saline and counted. Cell concentrations were adjusted for transplantation by tail vein injection, with 2 million BMCs in 300 µl 1640 medium per mouse. *Fah* ^-/-^ recipient mice were lethally irradiated with a dose of 14 Gy total body irradiation in a split dose with a 4-hour interval (200 cGy/min, 7Gy×2) the day before transplantation. Anti-AsGM1 (Wako Pure Chemicals Industries, Osaka, Japan) was administered (50 µg, *i.v.*) to deplete NK cells in recipient mice.

### Analysis of mice

Mice were monitored and body weight changes were recorded after cell transplantation. To obtain peripheral blood cells and plasma, mice were bled from the tail. When sacrificed, single cell suspensions from organs were prepared, and red blood cells were lysed. Liver mononuclear cells (MNCs) were prepared as described [21].

### Assay for metabolic parameters of mice

Serum biochemical markers, including alanine aminotransferase (ALT), aspartate aminotransferase (AST), alkaline phosphatase (ALP), gamma-glutamyl transpeptidase (GGT), total bilirubin (TBILI), creatinine (CRE), and albumin (ALB), were assessed by the standard photometric method using the biochemical detection kits (Rong Sheng, Shanghai, China) following the manufacturer's instructions.

### Isolation of mouse hepatocytes

Mouse hepatocytes were isolated as described [22]. Briefly, mice were anesthetized by intraperitoneal injection of 30 µg/g body weight sodium pentobarbital. The portal vein was cannulated, and the liver was subsequently perfused with ethylene glycol tetraacetic acid (EGTA) solution and digested with 0.075% collagenase solution. Viable hepatocytes suspended in Dulbecco's Modified Eagle's Medium (DMEM) (Gibco, NY, USA) solution were separated by a 40% Percoll (Gibco, NY, USA) solution with centrifugation at 400×*g* for 10 min at 4°C.

### Flow cytometry assay

Single-cell suspensions were blocked and incubated with the indicated fluorescent monoclonal antibodies (mAbs). Samples were acquired by a BD FACScalibur cytometer and analyzed by FlowJo software. The anti-mouse mAbs used for flow cytometry included the following: anti-mouse CD16/CD32; FITC-conjugated anti-CD49b (DX5), anti-CD8 (H35-17.2), anti-H-2K^k^ (AF3-12.1); PE-conjugated anti-CD49b (DX5), anti-CD8 (H35-17.2), anti-H-2D^d^ (34-2-12), anti-H-2D^b^ (KH95); PerCP-conjugated anti-CD19 (ID3); PE-Cy^TM^7-conjugated anti-NK1.1 (PK136), anti-CD45 (30-F11); APC-conjugated anti-CD3 (145-2C11); and APC-Cy^TM^7-conjugated anti-CD4 (GK1.5). The anti-rat mAbs used for flow cytometry included: FITC-conjugated anti-RT1A (C3); PE-conjugated anti-CD161a (10/78); PerCP-conjugated anti-CD8α (OX-8); PE-Cy^TM^7-conjugated anti-CD4 (OX-35); and APC-conjugated anti-CD3 (1F4). Appropriate isotype-matched, irrelevant control mAbs were used to determine the level of background staining. All the antibodies were purchased from BD Biosciences (San Diego, CA, USA).

### Histological examination

Liver, spleen, and lymph node samples were fixed in 10% neutral-buffered formalin and embedded in paraffin. Sections of 6- µm thickness were affixed to slides, deparaffinized, dehydrated, and then stained with hematoxylin and eosin (H&E) using routine methods.

### Immunohistochemistry

Commercially available monoclonal antibodies to mouse CD3 (Abcam, Cambridge, UK), mouse CD19 (Abcam), EGFP (Clontech, Otsu, Japan), and a polyclonal antibody to FAH (Abnova, CA, USA) were used for immunohistochemistry staining. After heat- or protease-induced antigen retrieval, formalin-fixed and paraffin-embedded liver tissue sections were stained with primary antibodies overnight at 4°C. The slides were subsequently incubated with ImmPRES anti-rabbit Ig or anti-mouse Ig (Vector Laboratories, Burlingame, CA) at room temperature for 30 min, stained with peroxidase substrate 3, 3′-diaminobenzidine chromogen (Vector Laboratories), and finally counterstained with hematoxylin.

### Vaccination of transplanted mice

For HBV vaccination, mice were vaccinated intramuscularly with 1 µg recombinant HBsAg protein vaccine (Biokangtai Company, Shenzhen, China) twice at a two-week interval. For OVA immunization, mice received two vaccinations subcutaneously at a two-week interval with 5 µg OVA protein (Sigma, St. Louis, MO, USA) dissolved in complete Freund's adjuvant (Sigma). Blood samples were obtained 1 week after booster vaccination by tail bleeding, and sera were kept at -20°C until used.

### RIA and ELISA

Serum anti-HBs levels were assessed using corresponding radioimmunoassay (RIA) kits (Beijing North Institute of Biological Technology, China) according to the manufacturer's instructions. OVA-specific antibodies were detected by ELISA. In brief, stripwell flat bottom polystyrene plates (Corstar, NY, USA) were coated with 1 µg/µl OVA (Sigma) overnight at 4°C, and then the membranes were blocked by a 5% BSA solution in a 200 µl volume. After being washed, plates were filled with 100 µl mice sera at appropriate dilution ratios for 1 hour followed by incubation with an HRP-conjugated goat anti-mouse IgG (Boster, Wuhan, China). Antibody levels were visualized by TMB substrate (eBioscience, San Diego) and collected as OD450 values.

### Statistical analysis

The two-tailed unpaired Student's *t*-test or ANOVA was used for statistical analyses. The experimental data was expressed as mean ± SEM, and the data are representative of at least 3 independent experiments. p<0.05 was considered statistically significant.

## Results

### Syngeneic BMT rescure *fah* ^-/-^ mice from liver failure by BM-derived FAH^+^ hepatocytes

The *fah* ^-*/*-^ mouse is a useful animal model for liver regeneration. Without administering a chemical (2-(2-nitro-4-trifluoromethylbenzoyl) cyclohexane-1, 3-dione, NTBC) to keep hepatocytes alive, *fah*-deficient hepatocytes die of accumulated toxic metabolites [23], and *fah* ^-*/*-^ mice therefore suffer from progressive liver failure and death (Figure S1).

**Figure 1:**
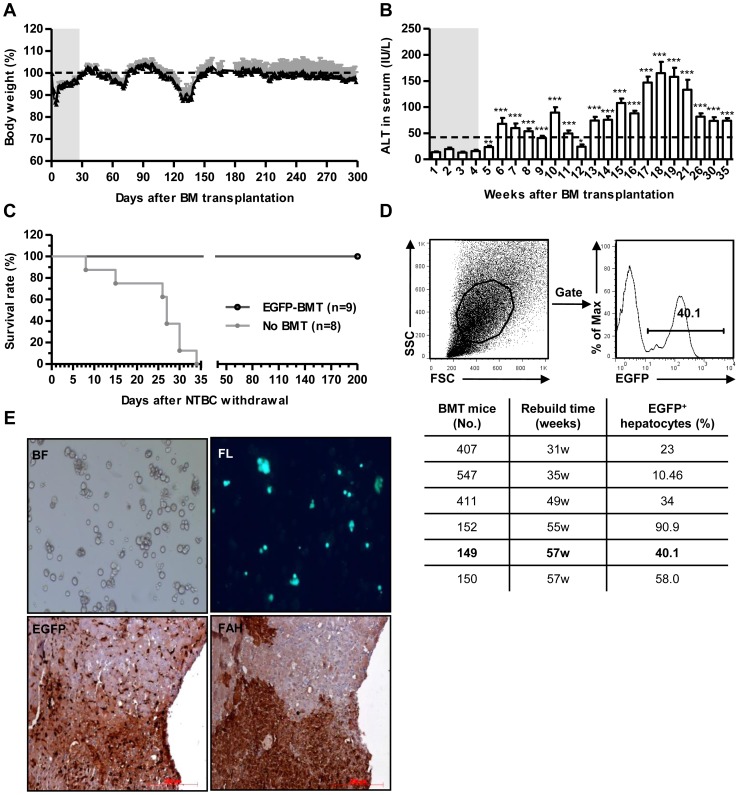
Syngeneic BMT rescure *fah^-/-^* mice from liver failure by BM-derived hepatocytes. (A-B) Body weight (A) and serum ALT (B) from recipient *fah^-/-^* mice after syn-BMT. NTBC was withdrawn on day 28 after BMT, and initial body weight was set as 100% (dotted line). Gray areas indicate NTBC administration, white areas indicate NTBC withdrawal. *p<0.05, **p<0.01, ***p<0.001. (mean ± SEM, n = 9). (C) Survival rate measurements from recipient *fah^-/-^* mice after NTBC withdrawal. No BMT *fah^-/-^* mice without NTBC treatment were set as a control. (D) EGFP^+^ hepatocytes from a BMT mouse by FACS (rebuild 57w). EGFP^+^ hepatocytes in other BMT mice are shown in the table below. (E) Donor-derived hepatocytes in BMT mouse 407. Freshly isolated mouse hepatocytes in bright field (left) and under fluorescence (right) are shown above, and EGFP (left) and FAH (right) immunohistochemistry in liver are shown below (brown staining indicates positive cells).

By carefully combining lethal irradiation and NTBC withdrawal in *fah* ^-*/*-^ mice, space is created in both the bone marrow (BM) and in the liver compartments in recipient mice. To determine whether BMCs from a single syngeneic donor can dually reconstitute the immune and hepatic system in recipient *fah* ^-*/*-^ mice after syn-BMT, we transplanted BM from *EGFP-Tg* mice (haplotype H-2^b^, *fah^+/+^*) into *fah* ^-*/*-^/129SvJ (H-2^b^) recipients. Though transplanted mice initially lost approximately 15% of their body weight, they soon recovered and maintained 100% of their initial weight for 150 days (Fig. 1A). Serum alanine aminotransferase (ALT) levels gradually increased after NTBC withdrawal and finally reduced to 80 IU/L, which differed from *fah* ^-*/*-^ mice without NTBC feeding (Fig. 1B and Figure S1C). All BMT mice survived at least 5 months after NTBC withdrawal beginning at day 28 (Fig. 1C). So syngeneic BMT could reduce liver injury and protect recipient *fah* ^-*/*-^ mice from death.

To evaluate hepatic reconstitution, long-term survivors were sacrificed and analyzed. Hepatocytes from BMT mice partially expressed EGFP, suggesting that they provided the neccessary FAH to restore liver function in recipient *fah* ^-*/*-^ mice (Fig. 1D). EGFP^+^ hepatocytes, which grow green under fluorescent microscope, were also found in freshly isolated hepatocytes. Liver histology revealed that the EGFP- and FAH-positive cells were organized into a cell cluster, reflecting a gradual reconstitution (Fig. 1E).

### Syngeneic BMT reconstitutes a functional immune system in recipient *fah^-/-^* mice

**Figure 2:**
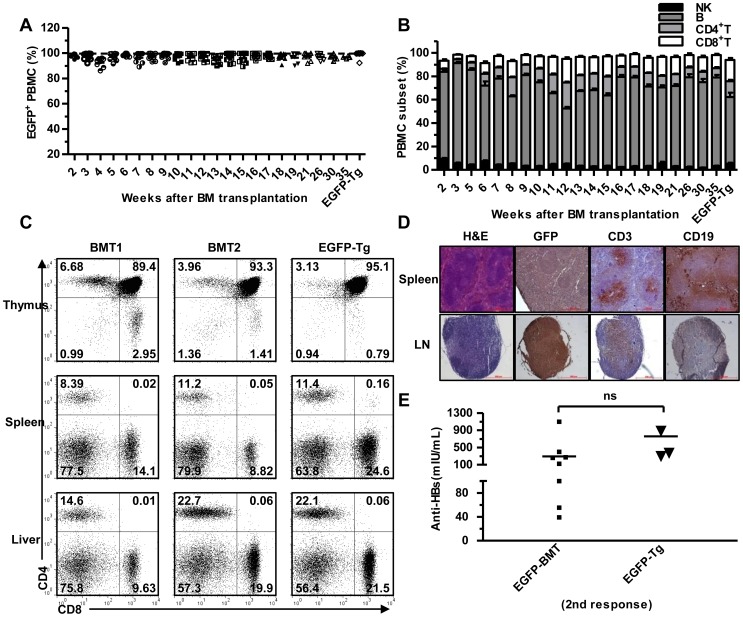
Immune reconstitution in recipient *fah^-/-^* mice after syngeneic BMT. (A) Donor-derived EGFP^+^ PBMC measurements from BMT mice at the indicated time points. (B) PBMC subset (NK, B, CD4^+^, and CD8^+^ T) measurements from BMT mice at the indicated times. PBMC from EGFP-Tg mice was set as a positive control. (mean ± SEM, n = 9). (C) Thymus, spleen, and liver CD4^+^ or CD8^+^ T lymphocyte detection in BMT mice (rebuild 9w) or *EGFP-Tg* donor mice by FACS. (D) Spleen and inguinal LN histology of serial sections of BMT mice (rebuild 9w). From L-R, H&E stain, EGFP, CD3, and CD19 immunohistochemistry. Brown staining indicates positive cells. (Original magnification 100×). (E) Serum anti-HBsAg levels in *EGFP-BMT* and *EGFP-Tg* mice after twice HBV vaccine immunizations.

Surviving recipients also showed stable multi-lineage hematopoietic reconstitution after syn-BMT. Nearly 100% of the PBMCs from the chimeras expressed EGFP, similar to the PBMCs from *EGFP-Tg* mice (Fig. 2A), and there was little difference in erythroid and lymphoid development between the chimeras and normal mice (Figure S2). PBMC subsets monitored at the indicated time points showed that NK, B, CD4^+^, and CD8^+^ T cells reconstituted normally in chimeras (Fig. 2B). Furthermore, we examined lymphocytes in thymus, spleen, and liver at week 9 after BMT and found a similar ratio of T cell subsets between syn-BMT and donor mice in all immune organs (Fig. 2C). Spleen and inguinal lymph node (LN) histology further confirmed successful immune reconstitution in BMT mice. Spleens from chimeras possessed white pulp structures, containing central arterioles surrounded by T and B cells. In some cases, typical germinal center formation was observed in both spleen and LN (Fig. 2D). To directly test the immune response after reconstitution, chimeric mice were immunized twice with an HBV vaccine. All recipient mice produced specific anti-HBsAg antibodies in serum, similar to donor *EGFP-Tg* mice (Fig. 2E), indicating there is a functional immune response. Together, these data suggest that BMCs from syngeneic donors can concurrently reconstitute both the immune and hepatic systems in recipient *fah^-/-^* mice.

### Allogeneic BMC reconstitutes a dual immune and hepatic system in recipient *fah^-/-^* mice

**Figure 3:**
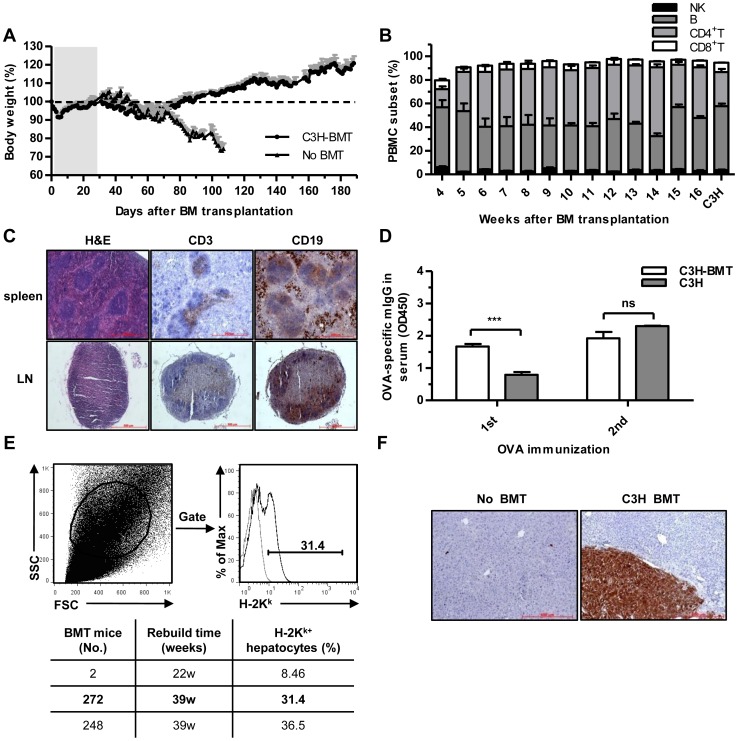
Immune and hepatic reconstitution in recipient *fah^-/-^* mice after allogeneic *C3H-BMT*. (A) Body weight in recipient *fah^-/-^* mice after allo-BMT. NTBC was withdrawn on day 28 after BMT, and initial body weight was set as 100% (dotted line). No BMT *fah* ^-/-^ mice without NTBC treatment were set as controls. Gray areas indicate NTBC administration, white areas indicate NTBC withdrawal. (mean ± SEM, n = 9). (B) PBMC subsets (NK, B, CD4^+^, and CD8^+^ T) from BMT mice over time. Donor C3H mice's PBMC was set as a positive control. (mean ± SEM, n = 5). (C) Spleen and inguinal LN histology of serial sections in BMT mice (rebuild 16w). From L-R: H&E stain, CD3, and CD19 immunohistochemistry. Brown staining indicates positive cells. (Original magnification 100×). (D) Serum anti-OVA levels from *C3H-BMT* and C3H mice after 1 or 2 OVA protein immunizations. (mean ± SEM, n = 4, ns: p>0.05, ***p<0.001). (E) Gating and MHC class I expression in hepatocytes from BMT mice (rebuild 39w) by FACS. Gray line indicates isotype control. H-2K^k+^ hepatocytes in other BMT mice are shown in the table below. (F) FAH immunohistochemistry in BMT mice (rebuild 16w). Liver section from No BMT *fah* ^-/-^ mice was set as a control. Brown staining indicates FAH*^+^* cells.

After successful liver and immune reconstitution in syn-BMT *fah^-/-^* mice, we next tested whether BMT using allogeneic donors would also concurrently reconstitute in *fah^-/-^* recipients. We transplanted BM from C3H/HeJSlac mice (H-2^k^, *fah^+/+^*) into *fah^-/-^*/129SvJ (H-2^b^) recipients. Approximately 60% of the allo-BMT mice survived for at least 5 months after NTBC withdrawal. Although these mice experienced an initial body weight loss of 10%, they soon recovered and maintained normal body weight for at least 150 days, whereas no-BMT mice lost their body weight day by day, and finally died when their body weight was less than 70% of the initial weight (Fig. 3A). Surviving recipients also exhibited stable multi-lineage hematopoietic reconstitution after allo-BMT, as nearly 100% of the PBMCs in the chimeras were H-2K^k+^, similar to C3H donors (Figure S3A), and there was little difference in total erythroid and lymphoid cell counts between chimeras and normal *fah^-/-^* mice (Figure S3B–C). Furthermore, the relative percentages of NK, B, CD4^+^, and CD8^+^ T cells subsets in PBMCs became normal over time (Fig. 3B). Spleen and inguinal LN histology further confirmed successful immune reconstitution in allo-BMT mice (Fig. 3C). The reconstituted immune response was functional in allo-BMT mice, as they produced specific anti-OVA antibodies in serum after immunization with OVA protein, similar to donor C3H mice (Fig. 3D).

Hepatocytes from allo-BMT mice also partially expressed donor MHC class I antigen (H-2K^k+^) (Fig. 3E), suggesting liver allo-repopulation with C3H mice's BM cells. Liver histology further showed that FAH^+^ hepatocytes were detected and organized in a cell cluster (Fig. 3F). From the above, allo-BMT successfully reconstituted both the immune system and hepatocytes in recipient *fah* ^-/-^ mice, indicating that chimeras with an MHC-matched immune system and liver from a single allogeneic donor can be created.

### Xenogeneic rat BMC concurrently reconstitutes their own immune and hepatic system in recipient *fah^-/-^* mice

**Figure 4:**
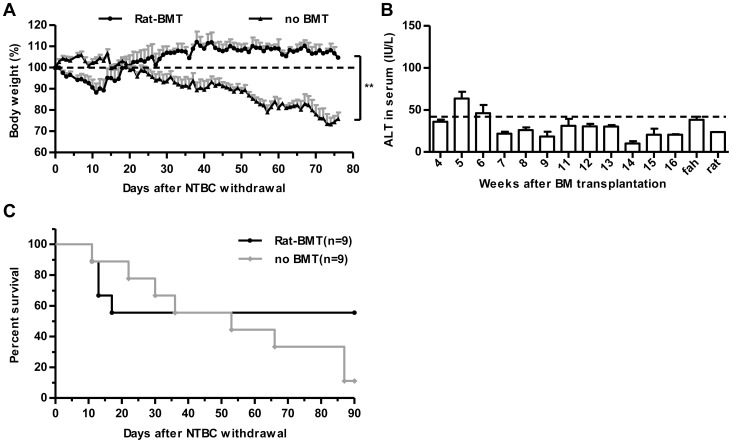
Xenogeneic BMT rescure *fah^-/-^* mice from liver failure and death. (A–B) Body weight (A) and serum ALT (B) from recipient *fah^-/-^* mice after xeno-BMT. NTBC was withdrawn on day 28 after BMT, and initial body weight was set as 100% (dotted line). **p<0.01. (mean ± SEM, n = 9). (C) Survival rate measurements from recipient *fah^-/-^* mice after NTBC withdrawal. No BMT *fah^-/-^* mice without NTBC treatment were set as controls.

**Figure 5:**
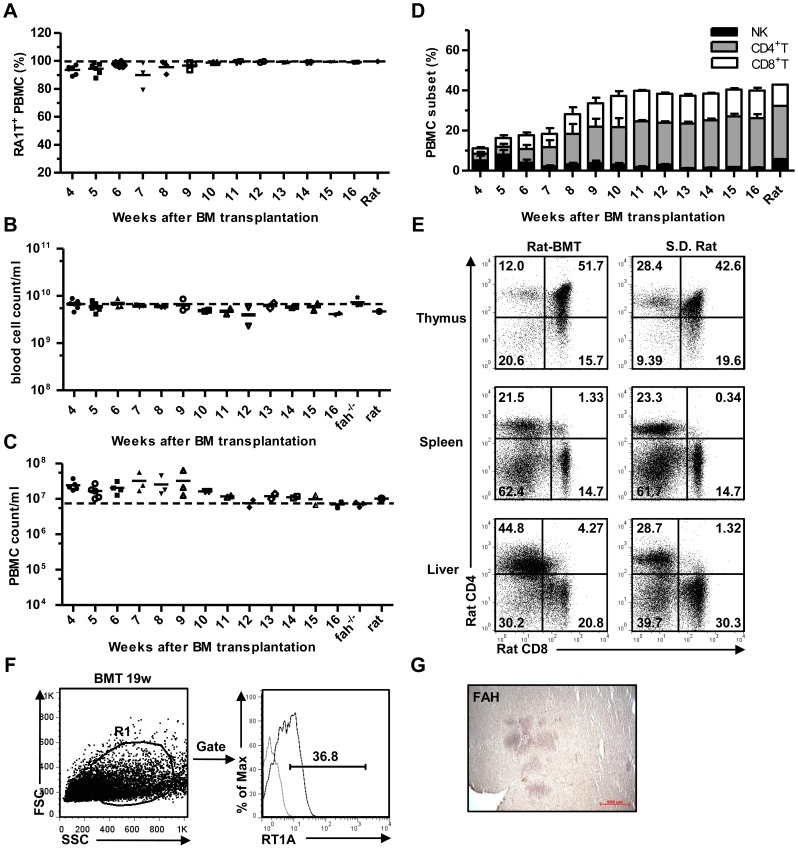
Immune and hepatic reconstitution in recipient *fah^-/-^* mice after xenogeneic BMT. (A) Donor-derived PBMC (RT1A^+^) measurements from BMT mice at the indicated time points. PBMC from S.D. donor rat was set as a positive control (dotted line). (mean, n = 5). (B–C) Blood cell (B) and PBMC (C) cellularity in peripheral blood from *rat-BMT* mice at the indicated time points. Normal *fah^-/-^* mice and S.D. rats were set as positive controls (dotted line). (mean, n = 5). (D) PBMC subset (NK, CD4^+^, and CD8^+^ T) measurements from BMT mice at the indicated time points. PBMC from SD donor rats was set as a positive control. (mean ± SEM, n = 5). (E) Thymus, spleen, and liver CD4^+^ or CD8^+^ T lymphocytes in xeno-BMT mice (rebuild 11w) or donor rats. (F) Gating and MHC class I expression in hepatocytes from BMT mice (rebuild 19w) by FACS. Gray line indicates isotype control. (G) FAH immunohistochemistry in BMT mice (rebuild 19w). Brown staining indicates FAH^+^ cells.

We next attempted to define whether xenogeneic BMC could successfully reconstitute the immune and hepatic system in *fah* ^-*/*-^ mice. We transplanted Sprague-Dawley rat (RT1A, *fah^+/+^*) BM into *fah^-/-^*/129SvJ (H-2^b^) recipients. Approximately 50% of xeno-BMT mice survived for at least 5 months after NTBC withdrawal, and serum ALT levels were maintained (Fig. 4). Surviving recipients also showed stable multi-lineage hematopoietic reconstitution. Nearly 100% of the PBMCs were RT1A^+^ in chimeras, and there was little difference in erythroid and lymphoid cell counts between chimeras and recipient mice, NK, CD4^+^, and CD8^+^ T cells in peripheral blood reconstituted normally over time (Fig. 5A–D). Furthermore, we found a similar ratio of T cell subsets in thymus, spleen, and liver between xeno-BMT mice and donor rat at week 11 after BMT (Fig. 5E). Anti-RT1A antibody staining demonstrated that hepatocytes from rat-BMT mice were partially positive for donor MHC class I antigen (Fig. 5F), suggesting that rat BM-derived hepatocytes were generated. Liver histology also showed that FAH^+^ cells were organized in a cell cluster (Fig. 5G). Taken together, BMC from rat could indeed reconstitute both immune and hepatic systems in *fah^-/-^* mice.

### BM-derived hepatocytes resulted from fusion between donor BM-derived myelomonocytic cells and host hepatocytes

**Figure 6:**
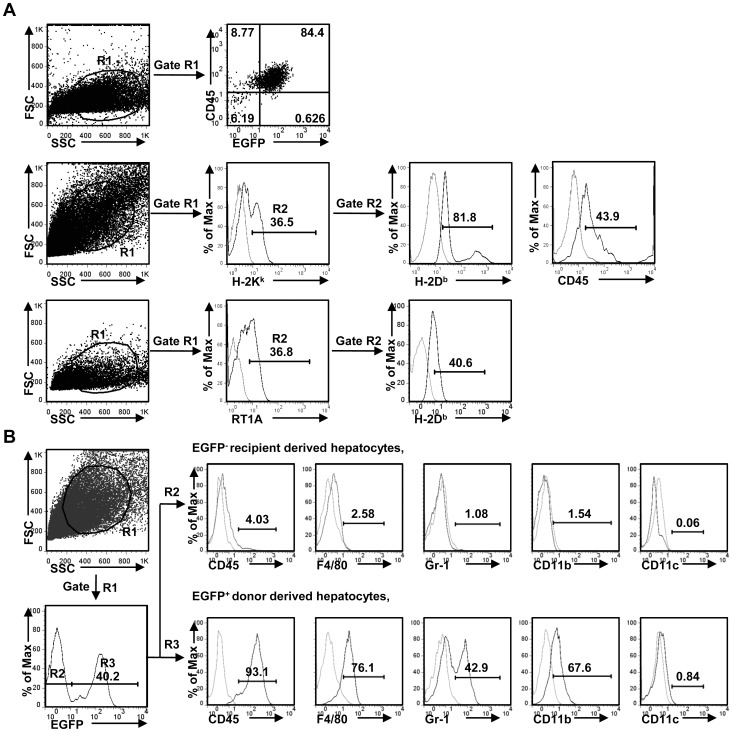
BM-derived hepatocytes emerged from fusion between donor BM-derived myelomonocytic cells and host hepatocytes. (A) Donor-derived hepatocyte measurements from *EGFP-BMT* (top), *C3H-BMT* (middle), and *rat-BMT* mice (bottom). Gated donor-derived hepatocytes mostly expressed CD45, and the same hepatocytes were partially positive for recipient MHC class I antigen. EGFP^+^, H-2K^k+^, or RT1A^+^ hepatocytes represent donor antigen, while H-2D^b+^ represent recipient MHC antigen. (B) Immunophenotyping of bone marrow-derived hepatocytes in *EGFP-BMT* mice. Recipient derived hepatocytes (R2) were negative for CD45, F4/80, Gr-1, CD11b, CD11c staining, while donor derived hepatocytes (R3) were mostly positive for CD45, F4/80, Gr-1, and CD11b, but negative for CD11c. (Numbers indicate the percentage of each population).

Finally, we investigated whether BM-derived hepatocytes were generated by *in vivo* fusion process between liver infiltrating hematopoietic cells and host hepatocytes. As shown in Fig. 6A, donor MHC class I antigen and recipient MHC class I antigen as well as CD45 antigen were concurrently expressed on BM-derived hepatocytes, suggesting cellular fusion occurred between BM-derived myelomonocytic progenitors and resident hepatocytes, which is known to occur after BMT [19]. Further analysis showed that EGFP^+^ donor derived hepatocytes were mostly CD45^+^F4/80^+^Gr-1^+^CD11b^+^CD11c^-^, while EGFP^-^ recipient derived hepatocytes were negative for all the above markers (Fig. 6B). Thus, after BM transplantation, BMC might differentiate into CD11b^+^ myelomonocytic progenitors and entry into the liver, and then fuse with host hepatocytes under the selection pressure.

### Metabolic parameters of transplanted animals

**Figure 7:**
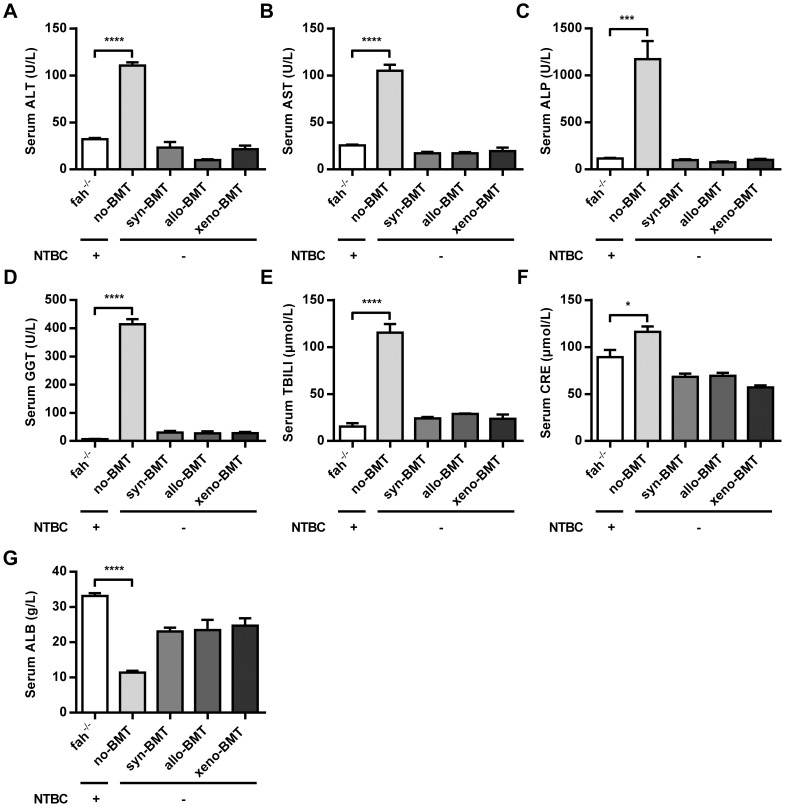
Analysis of metabolic serum parameters. Serum samples, collected from *EGFP-BMT* (syn-), *C3H-BMT* (allo-), and *rat-BMT* (xeno-) mice 20 weeks after NTBC withdrawal, were assessed. *Fah^-/-^* mice with NTBC on and off (4 weeks after NTBC withdrawal) were set as controls. (A) Alanine aminotransferase (ALT, U/L). (B) Aspartate aminotransferase (AST, U/L). (C) Alkaline phosphatase (ALP, U/L). (D) Gamma-Glutamyl Transpeptidase (GGT, U/L). (E) Total bilirubin (TBILI, µmol/L). (F) Creatinine (CRE, µmol/L). (G) Albumin (ALB, g/L). *p<0.05, ***p<0.001, ****p<0.0001. (mean ± SEM, n = 5).

Additionally, we assessed serum biochemical markers for *EGFP-BMT* (*syn-*), *C3H-BMT* (*allo-*), and *rat-BMT* (*xeno-*) mice 20 weeks after NTBC withdrawal, and compared with levels in *fah^-/-^* mice with NTBC on and off. As shown in Fig. 7, after NTBC withdrawal, no-BMT *fah^-/-^* mice showed a significantly increase in serum ALT, AST, ALP, GGT, TBILI, CRE, with a largely decrease in serum ALB, which indicated the impaired hepatocytes metabolism. However, *syn/allo/xeno-BMT* mice showed a substantial improvement in all tested parameters to levels near that found in NTBC on *fah^-/-^* mice. Thus, metabolic parameters of BM-derived hepatocytes resembled those of NTBC on *fah^-/-^* mice.

## Discussion

In summary (Table 1), our major finding is that BMCs from a single individual can successfully differentiate into both immune cells and hepatocytes, and functionally repopulate the immune system and liver in irradiated *fah^-/-^* mice. From our knowledge, it is the first mouse model with immune and liver reconstitution from a single BMC donor across the host species barrier, which was carried out by using syngeneic, allogeneic, or xenogeneic BMT. These findings also provide a further approach for establishing a dual humanized mouse model with HLA-matched human immune cells and human hepatocytes by using a more immunodeficient *fah^-/-^* mouse, such as *fah^-/-^ NOG*, in the future.

**Table:** **Table 1.** Donor MHC-restricted dual reconstitution of immune system and liver across species in mouse.

Transplantation Type	Donor (MHC haplotype)	Recipient (MHC haplotype)	Survival after NTBC withdrawal^1^	Immune reconstitution^2^	Hepatic reconstitution	
**No-BMT, ** ***fah^-/-^*** ** host**	No	129s4 (H-2^b^)	No	No	No	
**Syn-BMT, ** ***fah^-/-^*** ** host**	EGFP-Tg (H-2^b^)	129s4 (H-2^b^)	ALL (45/45)	Yes	31-35w	10-23%
					49w	∼34%
					55-57w	40-90%
**Allo-BMT, ** ***fah^-/-^*** ** host**	C3H/HeJSlac (H-2^k^)	129s4 (H-2^b^)	Partial (16/27) 3	Yes	22w	∼8.5%
					39w	31-36%
**Xeno-BMT, ** ***fah^-/-^*** ** host**	Rat (RT-1)	129s4 (H-2^b^)	Partial (10/36) 4	Yes	19w	∼36.8%

1 Survivors in total bone marrow transplanted (BMT) animals for at least 4 months after NTBC withdrawal.

2 BMT animals exhibited donor-derived immune cells and organ structure, and could respond to antigen immunization.

3 7 in 27 *allo-BMT* mice died from GVHD in the first 28 days before NTBC withdrawal, only 4 mice died after NTBC cutoff.

4 17 in 36 rat-BMT mice died in the first 28 days before NTBC withdrawal, and 9 in the rest 19 mice died after NTBC cutoff.

Development of humanized mice provides insights into *in vivo* human biology, which would otherwise be severely limited by ethical and/or technical constraints. Human immune system (HIS) mice are already established, showing a potential as the available model for the study of human immune response and human lymphotropic pathogens in mice [24]–[26], and human liver chimeric mice were developed for study of human hepatotropic pathogens [27]–[29] or preclinical evaluation of anti-hepatitis virus drug candidates [30]. However, further investigation of the pathology, immune correlates, and mechanisms of highly specialized pathogens like HBV, HCV and malaria (at liver stage) needs an excellent mouse model engrafted with MHC-restricted human immune system and pathogen-targeting organs. Recently, AFC8-hu HSC/Hep mice model was developed by meeting this requirement through co-implantation of human CD34^+^ HSCs and hepatocyte progenitor cells from a 15–18 weeks old fetal liver tissue into BALB/c-*Rag2* ^-/—^ *Il2rγ* ^-/-^ mice [31]. Although this approach successfully provides immune system and liver cells together in recipients, its extensive utilization is limited by obtaining human fetal liver tissues. In our model, syngeneic, allogeneic and xenogeneic BMT mice may reconstitute the immune system and liver from donor's BMCs, and based on these findings, it is possible that a more practical dual humanized mouse (e.g.HIS/HuHep mice) could be created in near future. Like AFC8-hu HSC/Hep mice model, HBV or HCV could infect and replicate in HIS/HuHep mice's liver, more similar to that of HBV or HCV clinical infection.

Despite its advantages, further improvements of our model can be envisioned. Firstly, it took a long time to create such a model, typically more than 20 weeks. Perhaps HSC differentiated slowly *in vivo*, *in vitro* cultivation the HSCs into hepatic progenitor cells and hematopoietic progenitor cells before transplantation may partially solve this problem. However, HSCs *ex vivo* cultivation then became another key challenge for us. Secondly, we observed that hematopoietic stem cells generated hepatocytes by cell fusion at a low frequency, which are in agreement with the former studies [9]. Macrophage depletion using chemicals [32], cytokines [33], [34], HLA expression [35] or even immunosuppressive drugs [36] treatments may properly further refine our model. Thirdly, GVHD was major issue in *allo/xeno-BMT* mice. Actually, 17 in 36 rat-BMT mice died in the first 28 days after bone marrow transplantation but before NTBC withdrawal (NTBC was cut off on day 28), which were considered to die from GVHD. Purification HSC from bone marrow before transplantation may reduce the incidence of GVHD [9]. Administration of cyclophosphamide, OKT3, or Il-21 signaling inhibitor was also reported to ameliorate xenogeneic GVHD [37]–[39]. Finally, the background strain of the mice (129S4.B6) is not optimal for xenorepopulation [40]. Although further improvements of our model are likely, we successfully reconstituted donor hepatocytes and immune cells across host species barrier using *fah*-deficient mice and simple BMT. We took a key step to construct the ideal model, and provided experimental evidence for the next HIS/HuHep mice model development.

HSCs have been reported to generate hepatocytes [41], cardiac myocytes [42], skeletal muscle [43], gastrointestinal epithelium [44], endothelium [45], and nerve cells [46] *in vivo*. Our new technique would make “multi-tissue” humanized mouse [47] model available, provided that the recipient mouse has open tissue space for donor-cell replacement, similar to the *fah^-/-^* mice used here for hepatocyte. Such a tool will not only be invaluable to study MHC-restricted interaction between immune cells and their targeted organ/tissue from a single donor, but also critical to developing effective and affordable vaccines and other immunotherapeutic approaches.

## Supporting Information

Figure S1 ***Fah^-/-^*** **mice exhibit progressive liver failure and death unless treated with NTBC.** (A) Body weight measurements from mice with or without 2-(2-nitro-4-tifluoro-methylbenzyol)-1, 3-cyclohexanedione (NTBC) treatment for hepatocyte survival. Initial body weight was set as 100% (dotted line). ***p<0.001. (B–C) Survival rate (B) and serum ALT (**C**) measurements in mice with or without NTBC treatment. ns: p>0.05, **p<0.01, ***p<0.001. (mean ± SEM, n = 10).
(PDF)

Figure S2 **Blood cell and PBMC reconstitution in peripheral blood of recipient** ***fah^-/-^*** **mice after syngeneic** ***EGFP-BMT*** **.** (A–B) Blood cell (A) and PBMC (B) cellularity from the peripheral blood of *EGFP-BMT* mice. Normal *fah^-/-^* mice with NTBC treatment were set as the positive control (dotted line). (mean, n = 5).
(PDF)

Figure S3 **Blood cell and PBMC reconstitution in peripheral blood of recipient** ***fah^-/-^*** **mice after allogeneic** ***C3H-BMT*** **.** (A) Donor-derived PBMC (H-2K^k+^) measurements from BMT mice at the indicated time points. C3H donor mice were set as the positive control (dotted line). (B–C) Blood cell (B) and PBMC (C) cellularity from peripheral blood of *C3H-BMT* mice at the indicated time points. (mean, n = 4). Normal *fah^-/-^* mice with NTBC treatment were set as the positive control (dotted line).
(PDF)

File S1 **ARRIVE Checklist.**
(PDF)
