# Supplementary material for: Bone Marrow Transplantation Concurrently Reconstitutes Donor Liver and Immune System across Host Species Barrier in Mice
Source: PLoS One. 2014 Sep 5;9(9):e106791. doi: 10.1371/journal.pone.0106791 (PMC4156390; doi:10.1371/journal.pone.0106791)
Supplement: Figure S1 — Fah-/- mice exhibit progressive liver failure and death unless treated with NTBC. (A) Body weight measurements from mice with or without 2-(2-nitro-4-tifluoro-methylbenzyol)-1, 3-cyclohexanedione (NTBC) treatment for hepatocyte survival. Initial body weight was set as 100% (dotted line). ***p<0.001. (B–C) Survival rate (B) and serum ALT (C) measurements in mice with or without NTBC treatment. ns: p>0.05, **p<0.01, ***p<0.001. (mean ± SEM, n = 10). (PDF) [file pone.0106791.s001.pdf]

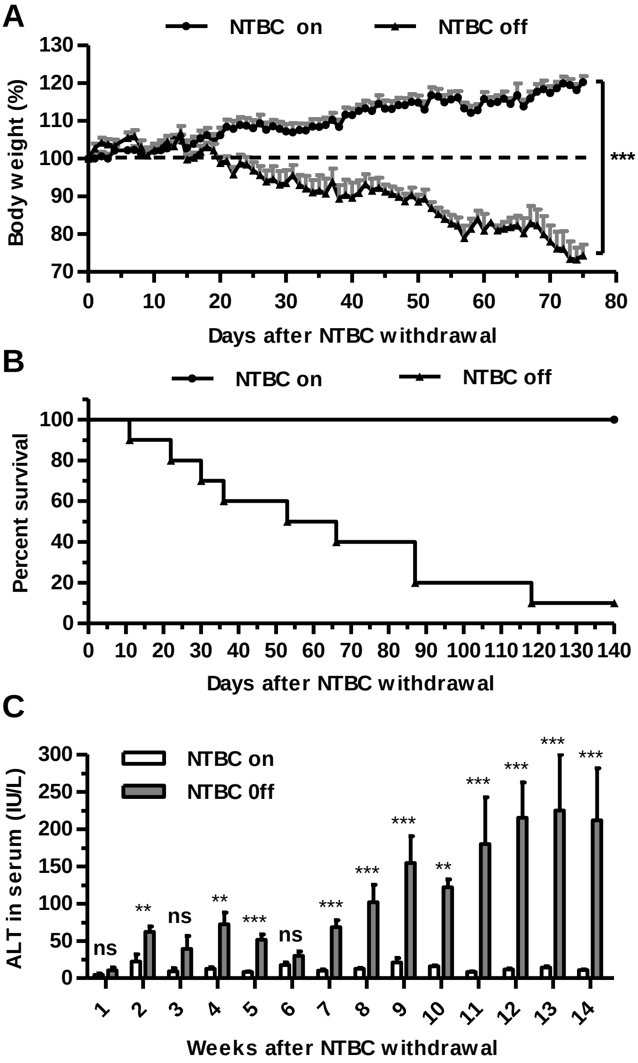

Figure S1: ***Fah***$^{-/-}$ **mice exhibit progressive liver failure and death unless treated with NTBC.** (**A**) Body weight measurements from mice with or without 2-(2-nitro-4-tifluoro-methylbenzyol)-1,3-cyclohexanedione (NTBC) treatment for hepatocyte survival. Initial body weight was set as 100% (dotted line). ***p<0.001. (**B-C**) Survival rate (**B**) and serum ALT (**C**) measurements in mice with or without NTBC treatment. ns: p>0.05, **p<0.01, ***p<0.001. (mean ± SEM, n=10).
