# Supplementary material for: Bone Marrow Transplantation Concurrently Reconstitutes Donor Liver and Immune System across Host Species Barrier in Mice
Source: PLoS One. 2014 Sep 5;9(9):e106791. doi: 10.1371/journal.pone.0106791 (PMC4156390; doi:10.1371/journal.pone.0106791)
Supplement: Figure S2 — Blood cell and PBMC reconstitution in peripheral blood of recipient fah-/- mice after syngeneic EGFP-BMT . (A–B) Blood cell (A) and PBMC (B) cellularity from the peripheral blood of EGFP-BMT mice. Normal fah-/- mice with NTBC treatment were set as the positive control (dotted line). (mean, n = 5). (PDF) [file pone.0106791.s002.pdf]

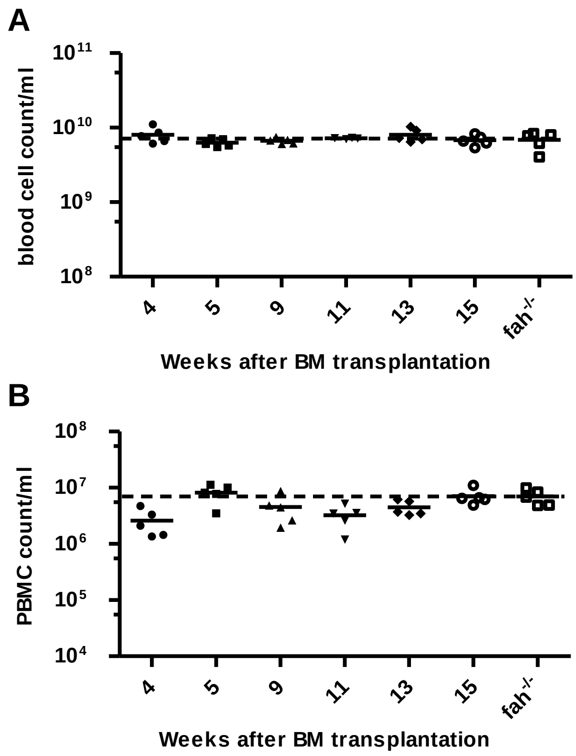

Figure S2: Blood cell and PBMC reconstitution in peripheral blood of recipient *fah*$^{-/-}$ mice after syngeneic *EGFP-BMT*. (**A-B**) Blood cell (**A**) and PBMC (**B**) cellularity from the peripheral blood of *EGFP-BMT* mice. Normal *fah*$^{-/-}$ mice with NTBC treatment were set as the positive control (dotted line). (mean, n=5).
