# Supplementary material for: Bone Marrow Transplantation Concurrently Reconstitutes Donor Liver and Immune System across Host Species Barrier in Mice
Source: PLoS One. 2014 Sep 5;9(9):e106791. doi: 10.1371/journal.pone.0106791 (PMC4156390; doi:10.1371/journal.pone.0106791)
Supplement: Figure S3 — Blood cell and PBMC reconstitution in peripheral blood of recipient fah-/- mice after allogeneic C3H-BMT . (A) Donor-derived PBMC (H-2Kk+) measurements from BMT mice at the indicated time points. C3H donor mice were set as the positive control (dotted line). (B–C) Blood cell (B) and PBMC (C) cellularity from peripheral blood of C3H-BMT mice at the indicated time points. (mean, n = 4). Normal fah-/- mice with NTBC treatment were set as the positive control (dotted line). (PDF) [file pone.0106791.s003.pdf]

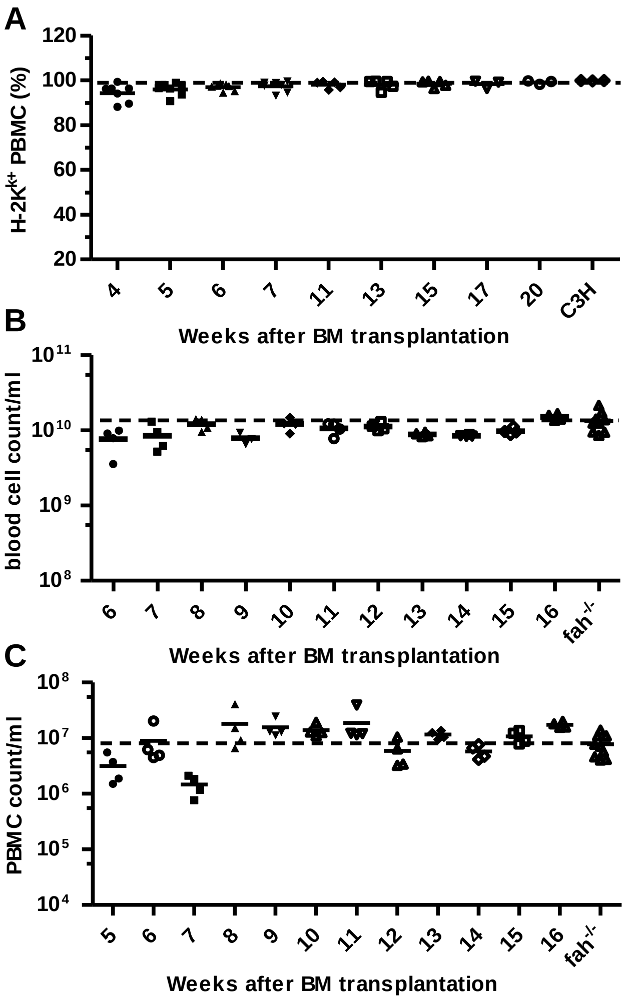

Figure S3: Blood cell and PBMC reconstitution in peripheral blood of recipient *fah*$^{-/-}$ mice after allogeneic *C3H-BMT*. (**A**) Donor-derived PBMC (H-2K^{k+}) measurements from BMT mice at the indicated time points. C3H donor mice were set as the positive control (dotted line). (**B-C**) Blood cell (**B**) and PBMC (**C**) cellularity from peripheral blood of *C3H-BMT* mice at the indicated time points. (mean, n=4). Normal *fah*$^{-/-}$ mice with NTBC treatment were set as the positive control (dotted line).
